# Postoperative superior mesenteric artery syndrome following appendicectomy: A case report^☆^

**DOI:** 10.1016/j.ijscr.2021.106629

**Published:** 2021-11-26

**Authors:** Ali Mohtashami, Juanita N. Chui, Cameron Law, Dane Cole-Clark, Robert Simon

**Affiliations:** a Lismore Base Hospital, Lismore, NSW, Australia; b Royal North Shore Hospital, Sydney, NSW, Australia

**Keywords:** Superior mesenteric artery syndrome, Duodenal obstruction, Aortomesenteric syndrome, Case report

## Abstract

**Introduction:**

Superior mesenteric artery syndrome is a rare cause of proximal intestinal obstruction. It is caused by a narrow aortomesenteric angle resulting in external compression of the duodenum as it traverses between the abdominal aorta and the superior mesenteric artery. Presenting symptoms tend to be non-specific and aetiological risk factors for this syndrome remain subjects to debate. The lack of awareness for this phenomenon often results in a delayed diagnosis, yet it can predispose to potentially life-threatening complications.

**Case presentation:**

We describe an acquired case of SMA syndrome, in an 88-year old male who underwent an open appendicectomy 20 years prior. The patient presented with an 18-month history of progressive anorexia, weight loss, and intractable vomiting. After inconclusive initial investigations, an exploratory laparotomy demonstrated extensive postoperative adhesions, placing traction on the SMA through its ileocolic branch, resulting in acute angulation of the SMA and subsequent external duodenal compression.

**Discussion:**

This case illustrates the acute evolving presentation of gastric and duodenal obstruction associated with SMA syndrome, and the need to raise the index of suspicion for its diagnosis. In this case, it is postulated that SMA syndrome presented as a late complication of an open appendicectomy - A rare presentation for a rare disease.

**Conclusion:**

SMA syndrome is an uncommon, but important differential for upper gastrointestinal obstruction. This case illustrates the challenges in the diagnosis of this rare clinical entity. Further study is warranted to understand the varied aetiology and optimal management for patients presenting with SMA syndrome.

## Highlights

• Superior mesenteric artery syndrome is a rare, but important cause of proximal intestinal obstruction.
• It is postulated that postoperative adhesions can result in superior mesenteric artery syndrome.
• The lack of awareness for this phenomenon can result in a delayed diagnosis and predispose to life-threatening complications.

## 1 Introduction

Superior mesenteric artery (SMA) syndrome is a rare cause of proximal intestinal obstruction. It is characterised by extrinsic compression of the third portion duodenum between the abdominal aorta and the SMA at its origin. It has been described in the literature by a variety of other names, including Cast syndrome, Wilkie syndrome, arteriomesenteric duodenal obstruction, and chronic duodenal ileus [1], [2]. This rare, but potentially life-threatening syndrome often presents a diagnostic challenge where symptoms do not always correlate well with radiologic studies [3], [4].

This case has been reported in line with the SCARE Criteria 2020 [5].

## 2 Case presentation

An 88-year-old male presented to a base hospital in rural NSW, Australia, with intractable vomiting, associated with an 18-month history of anorexia and unintentional weight loss of 15 kg. This was on a background of idiopathic pulmonary fibrosis and an open appendicectomy 20 years prior. The patient reported no significant family history.

On examination, the patient was vitally stable and afebrile. Abdominal examination demonstrated epigastric distension and mild abdominal tenderness, with no evidence of peritonism. Initial biochemical investigations revealed a lactate level of 3.3 mmol/L and were otherwise unremarkable.

**Fig. 1:**
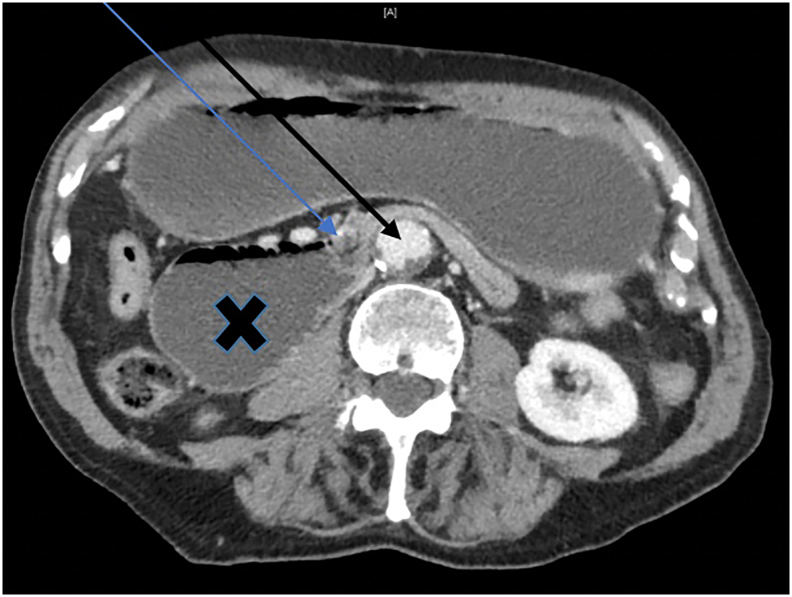
Computed tomography Axial view demonstrating extensive dilatation of proximal duodenum marked by X, between SMA and AAA marked by blue and black arrow respectively.

**Fig. 2:**
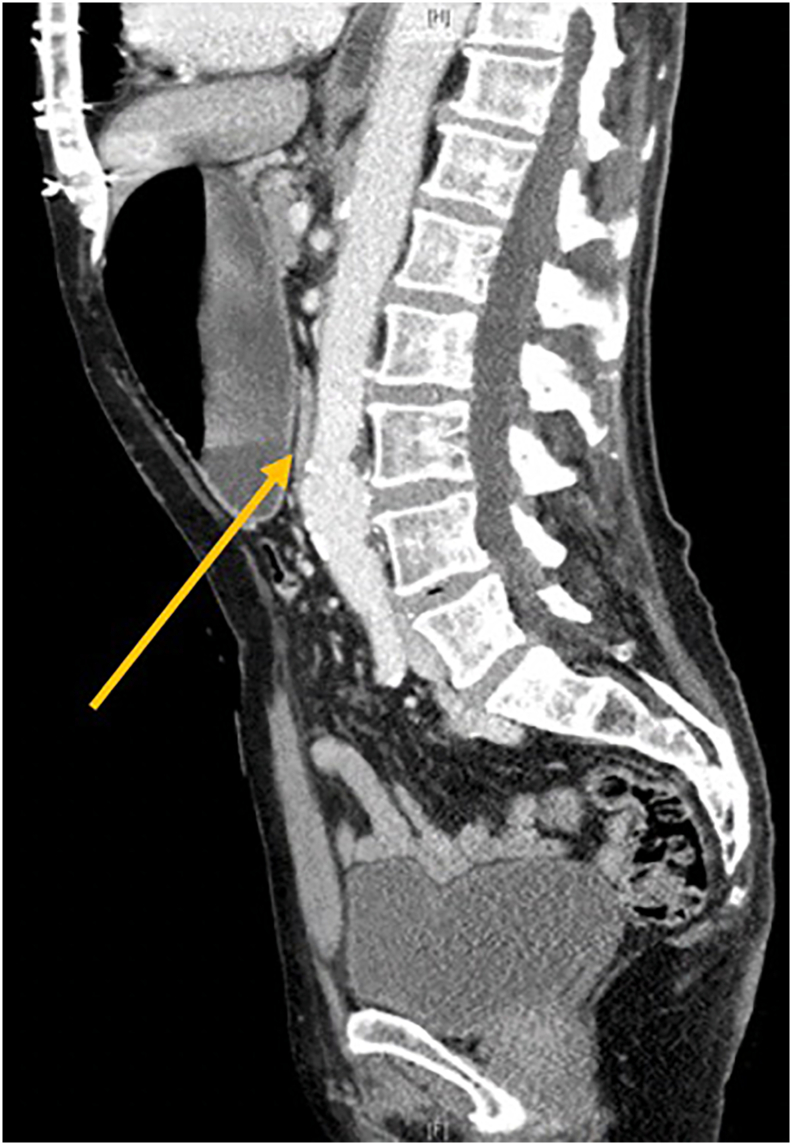
Computed tomography, sagittal view demonstrating collapsed in the 4th part of duodenum (indicated by arrow).

Abdominal computed tomography (CT) performed on admission revealed marked dilatation of the stomach and proximal duodenum, which were primarily fluid-filled (Fig. 1). A distinct transition point was noted at the third portion of the duodenum (D3) with collapse distal to this (Fig. 2). No clear cause could be identified for the obstruction.

**Fig. 3:**
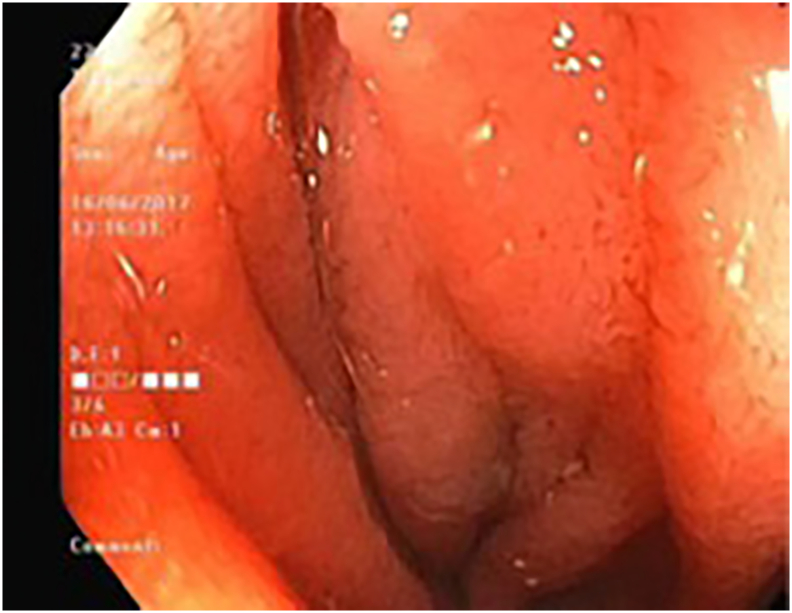
Endoscopic view of the narrowest point of 3rd portion of the duodenum.

The patient proceeded to an upper endoscopy, which revealed a short extrinsic stenosis at D3 (Fig. 3). This segment was traversed and no overt intraluminal pathology was identified, raising suspicion for SMA syndrome. The patient was consented for an exploratory laparotomy and potential gastro-jejunal bypass.

Intraoperative findings confirmed dilatation of the duodenum proximal to the overlying SMA, followed by abrupt constriction of the duodenum. In addition, a small abdominal aorta aneurysm, measuring 2.5 cm, was observed directly posterior to this. Further to this, extensive adhesions were found in the right iliac fossa, consistent with a previous appendicectomy, placing the ileocolic pedicle under tension and resulting in the impingement of the duodenum between the SMA and abdominal aorta.

Extensive adhesiolysis was performed over the right side of colon and terminal ileum, successfully releasing the ileocolic pedicle. The duodenal compression was relieved. There was no requirement for bowel resection. The patient recovered with no major complications. There have been no recurrence of symptoms on follow-up.

## 3 Discussion

This case describes an unreported mechanism of acquired SMA syndrome in an elderly patient, due to peritoneal adhesions in the context of remote open appendicectomy. It is postulated that this combined with an abdominal aortic aneurysm led to progressive narrowing of the aortomesenteric angle and the entrapment of the duodenum.

Within the existing literature, an estimated incidence of less than 0.3% has been reported for this rare entity [6]. Yet, with potentially life-threatening complications it represents an important differential for gastrointestinal obstruction. Normally, the aortomesenteric angle ranges from 38 to 56 degrees and produces a distance of 10-28 mm between the abdominal aorta and SMA, allowing the third portion of the duodenum to traverse posteriorly the SMA towards the ligament of Treitz without compression. SMA syndromes arise with an aortomesenteric angle of less than 25 degrees, corresponding to a distance of less than 10 mm [7].

Congenital and anatomical factors predisposing for the syndrome are well-recognised in the literature [8], [9]. This includes high insertion of the duodenum, low SMA origin, and short ligament of Treitz. Congenital compression has also been described due to Ladd bands in the context of midgut malrotation. This may be exacerbated by acquired factors, including rapid weight loss resulting in the loss of retroperitoneal fat, prolonged bed rest, retroperitoneal tumours, and abdominal trauma. To the best of our knowledge, this case presents a mechanism for acquired SMA syndrome has not been previously described in the literature.

Owing the diversity of etiological factors, the non-specific symptoms with which it often presents, and its rarity, the diagnosis of SMA syndrome can be challenging and often made by exclusion after extensive work-up. Delays in diagnosis can predispose to potentially life-threatening complications, such that a high index of suspicion for SMA syndrome is required when correlating between clinical and radiological findings.

## 4 Conclusion

This case describes an acquired SMA syndrome presenting as a late postoperative complication due to peritoneal adhesions, illustrating the complex aetiology of this syndrome and the challenges of its diagnosis. SMA syndrome represents a rare, but important differential for upper gastrointestinal obstruction.

## Provenance and peer review

Not commissioned, externally peer reviewed.

## Sources of funding

This research did not receive any specific grant from funding agencies in the public, commercial, or not-for-profit sectors.

## Ethical approval

No ethical approval was sought for this case report.

## Consent

Written informed consent was obtained from the patient for publication of this case report and accompanying images. A copy of the written consent is available for review by the Editor-in-Chief of this journal on request.

## CRediT authorship contribution statement

AM and JC gathered the data for this case and prepared the original manuscript. CL, DC, and RS reviewed the manuscript. AM, CL, DC, and RS were the operating surgeons in this case. All authors approved the final manuscript.

## Research registration

Not applicable.

## Guarantor

Dr. Ali Mohtashami is the guarantor and accepts full responsibility for the work and/or the conduct of the study, had access to the data, and controlled the decision to publish.

## Funding

This research did not receive any specific grant from funding agencies in the public, commercial, or not-for-profit sectors.

## Declaration of competing interest

The authors have no conflicts of interest to declare.

## References

[1] Dorph M.H. (1950). The cast syndrome: review of the literature and report of a case. N. Engl. J. Med..

[2] Gniftiths G.J., Whitehouse G.H., Wilkie D.P. (1921). Chronic duodenal ileus. Br. J. Surg..

[3] Cohen L.B., Field S.P., Sachar D.B. (1985). The superior mesenteric artery syndrome. The disease that isn't, or is it?. J. Clin. Gastroenterol..

[4] Hines J.R., Gore R.M., Ballantyne G.H. (1984). Superior mesenteric artery syndrome: diagnostic criteria and therapeutic approaches. Am. J. Surg..

[5] Agha R.A., Franchi T., Sohrabi C., Mathew G., Kerwan A., Thoma A., Beamish A.J., Noureldin A., Rao A., Vasudevan B., Challacombe B. (2020 Dec). The SCARE 2020 guideline: updating consensus surgical case report (SCARE) guidelines. Int. J. Surg..

[6] Welsch T., Buchler M.W., Kienle P. (2007). Recalling superior mesenteric artery syndrome. Dig. Surg..

[7] Mandarry M.T., Zhao L., Zhang C., Wei Z.Q. (2010). A comprehensive review of superior mesenteric artery syndrome. Eur. Surg..

[8] Merrett N.D., Wilson R.B., Cosman P. (2009). Superior mesenteric artery syndrome: diagnosis and treatment strategies. J. Gastrointest. Surg..

[9] Wyten R., Kelty C.J., Falk G.L. (2010). Laparoscopic duodenojejunostomy for the treatment of superior mesenteric artery (SMA) syndrome: case series. J. Laparoendosc. Adv. Surg. Tech. A.

